# Ginger Beer

**Published:** 1879-09

**Authors:** 


GINGER BEER.—Concentrated essence of Jamaica

| | |
|---|---|
| ginger - - - - - - - | 2 ounces. |
| The juice and rind of - - - - - | 4 lemons. |
| White sugar - - - - - - - | 3½ lbs. |
| Cream of tartar - - - - - - | 1 ounce. |
| Water - - - - - - - - | 1½ gallon. |
| Yeast - - - - - - | 1 tablespoonful |

To be kept in a warm place for about two days, strain, and bottle for use.

